# The Molecular Landscape of Primary Acral Melanoma: A Multicenter Study of the Italian Melanoma Intergroup (IMI)

**DOI:** 10.3390/ijms22083826

**Published:** 2021-04-07

**Authors:** Lisa Elefanti, Carolina Zamuner, Paolo Del Fiore, Camilla Stagni, Stefania Pellegrini, Luigi Dall’Olmo, Alessio Fabozzi, Rebecca Senetta, Simone Ribero, Roberto Salmaso, Simone Mocellin, Franco Bassetto, Francesco Cavallin, Anna Lisa Tosi, Francesca Galuppini, Angelo Paolo Dei Tos, Chiara Menin, Rocco Cappellesso

**Affiliations:** 1 Immunology and Diagnostic Molecular Oncology Unit, Veneto Institute of Oncology IOV-IRCCS, 35128 Padua, Italy; lisa.elefanti@iov.veneto.it (L.E.); stefania.pellegrini@iov.veneto.it (S.P.); 2 Anatomy and Histology Unit, Veneto Institute of Oncology IOV-IRCCS, 35128 Padua, Italy; carolina.zamuner@iov.veneto.it; 3 Soft-Tissue, Peritoneum and Melanoma Surgical Oncology Unit, Veneto Institute of Oncology IOV-IRCCS, 35128 Padua, Italy; paolo.delfiore@iov.veneto.it (P.D.F.); luigi.dallolmo@iov.veneto.it (L.D.); simone.mocellin@iov.veneto.it (S.M.); 4 Department of Molecular Medicine, University of Padua, 35128 Padua, Italy; camilla.stagni3@gmail.com; 5 Oncology Unit 3, Veneto Institute of Oncology IOV-IRCCS, 35128 Padua, Italy; alessio.fabozzi@iov.veneto.it; 6 Pathology Unit, Department of Oncology, University of Turin, 10124 Turin, Italy; rebesenetta@gmail.com; 7 Section of Dermatology, Department of Medical Sciences, University of Turin, 10124 Turin, Italy; simone.ribero@unito.it; 8 Pathological Anatomy Unit, Padua University Hospital, 35128 Padua, Italy; roberto.salmaso@aopd.veneto.it (R.S.); angelo.deitos@unipd.it (A.P.D.T.); rocco.cappellesso@gmail.com (R.C.); 9 Department of Surgery, Oncology and Gastroenterology (DISCOG), University of Padua, 35128 Padua, Italy; 10 Plastic Surgery Unit, Padua University Hospital, 35128 Padua, Italy; franco.bassetto@unipd.it; 11 Department of Neurosciences (DNS), University of Padua, 35128 Padua, Italy; 12 Independent Statistician, 36020 Solagna, Italy; cescocava@libero.it; 13 Pathological Anatomy Unit, AULSS5, Santa Maria della Misericordia Hospital, 45100 Rovigo, Italy; annalisa.tosi@aulss5.veneto.it; 14 Surgical Pathology Unit, Department of Medicine (DIMED), University of Padua, 35128 Padua, Italy; francesca.galuppini@gmail.com

**Keywords:** acral melanoma, *BRAF*, *NRAS*, *PREX2*, *ARID1A*, *KIT*, *TP53*, *TERT* promoter, copy number variations

## Abstract

Acral melanoma (AM) is a rare and aggressive subtype of melanoma affecting the palms, soles, and nail apparatus with similar incidence among different ethnicities. AM is unrelated to ultraviolet radiation and has a low mutation burden but frequent chromosomal rearrangements and gene amplifications. Next generation sequencing of 33 genes and somatic copy number variation (CNV) analysis with genome-wide single nucleotide polymorphism arrays were performed in order to molecularly characterize 48 primary AMs of Italian patients in association with clinicopathological and prognostic features. *BRAF* was the most commonly mutated gene, followed by *NRAS* and *TP53*, whereas *TERT* promoter, *KIT*, and *ARID1A* were less frequently mutated. Gains and losses were recurrently found in the 1q, 6p, 7, 8q, 20 and 22 chromosomes involving *PREX2*, *RAC1*, *KMT2C*, *BRAF*, *CCND1*, *TERT*, and *AKT3* genes, and in the 6q, 9, 10, 11q and 16q chromosomes including *CDKN2A*, *PTEN*, and *ADAMTS18* genes, respectively. This study confirmed the variety of gene mutations and the high load of CNV in primary AM. Some genomic alterations were associated with histologic prognostic features. *BRAF* mutations, found with a higher rate than previously reported, correlated with a low Breslow thickness, low mitotic count, low CNV of the AMs, and with early-stage of disease.

## 1. Introduction

Acral melanoma (AM) is a rare subtype of malignant melanoma that originates from the glabrous skin of the palms, soles, and nail apparatus (subungual) [1]. The more commonly affected sites are the great toe and the thumb [2]. Although its annual incidence is similar among the different racial groups worldwide, settling at about 1 case per 100,000 people, in proportion AM is relatively rare in the European-derived population but common in Asian, African, and Hispanic people [2,3]. Moreover, for this reason, most studies focused on these last ethnicities. AM prognosis is quite poor, with a five-year overall survival (OS) ranging from 59% to 70%, mainly as a consequence of delayed diagnosis due to the unusual localization and of the effect of the different histotypes and molecular backgrounds [4,5,6,7,8]. Patients with AM are typically diagnosed at an advanced stage of disease with thick and ulcerated primary tumor and a high likelihood of metastases [4,5,6,7,8]. The treatment is the same as for the other cutaneous melanomas: wide surgical removal of the primary tumor and, if feasible, of regional nodal metastases for local disease and immunotherapy or targeted therapy (more rarely chemotherapy) for metastatic disease [9]

From the pathological point of view, AM is classified as acral lentiginous melanoma (ALM), which is the most common and aggressive form, superficial spreading melanoma (SSM), nodular melanoma (NM), and nevoid melanoma [9,10,11,12,13,14]. These differ not only in morphology but also in etiology, pathogenesis, and underlying molecular alterations [10]. Compared to other non-acral cutaneous melanomas showing a high number of mutations induced by ultraviolet (UV) radiation, this appears to be a less important etiologic factor for AM since this type of tumor has a low mutation burden and several chromosomal rearrangements and gene amplifications [15,16,17,18,19,20,21,22,23]. The reported frequencies of gene mutations vary in the different studies and this is probably due to the low number of analyzed cases, the predominant use of metastatic material, and the inclusion of different histotypes of melanoma in the casuistries. *BRAF* mutations have been reported to occur at a significantly lower rate in AM than in other non-acral skin melanomas (about 10–20% of cases versus 50% of cases, respectively) [15,16,17,18,23]. Mutated *NRAS* and *KIT* have been detected in about 15% of cases, with a respective range of 7–47% and 6–21% among the studies [16,17,22,23,24,25,26,27,28]. *TP53* mutations have been reported as a rare and late event in AM [16,19,29]. Instead, mutations in *NF1* and amplification of *KIT*, *CCND1*, *PAK1*, *GAB2*, *CDK4*, *RICTOR*, and *TERT*, have been more commonly found in AM than in other skin melanomas [16,19,20,28]. 

This study aimed to characterize the molecular landscape of primary AM in an Italian cohort of patients and to assess the possible associations of molecular alterations with the clinicopathological features and the prognosis of patients.

## 2. Results

### 2.1. Patients’ Characteristics

Of the original series of 74 FFPE AM samples, 26 (19%) did not yield amplifiable DNA for molecular analysis. The remaining 48 patients (81%) with analyzable samples had a mean age of 71.2 ± 13.2 years (range 47–88 years) and a male to female ratio of 1:1. The clinicopathological features of patients are summarized in Table 1. Most AMs were located in the foot (79%), followed by nail (15%) and hand (6%). The most common histotype was acral lentiginous melanoma (83%), followed by nodular melanoma (13%), superficial spreading melanoma (2%), and nevoid melanoma (2%). The vast majority of AMs were locally advanced (T3 and T4 stages; 80%). Ulceration was a common feature, being present in 58% of cases. Regression and lymphovascular and perineural invasion were present in 8%, 32%, and 23% of AMs, respectively.

### 2.2. Molecular Classification

Forty AM cases were stratified into the four TCGA classification groups based on the genotyping analysis by a 33-gene next generation sequencing (NGS) custom panel (30 samples) and hotspot Sanger sequencing (10 samples). Eight cases failed sequencing analysis. A frequency of mutually exclusive mutations of 35% for *BRAF*, 28% for *NRAS*, and 5% for *NF1* genes was found; the remaining 32% of AMs were classified as triple wild-type (TWT). *BRAF* mutations were a p.V600E substitution in all cases but one (93%) with p.V600D substitution. *BRAF* mutations were found in all the four histotypes, with a frequency of 32% in acral lentiginous melanoma. *NRAS* mutations were missense in the p.Q61 codon for 8 cases (3 p.Q61R, 2 p.Q61K, 2 p.Q61H, and 1 p.Q61L) or the p.G12 codon for 3 cases (2 p.G12D and 1 p.G12C cases). These last mutations were found in two acral lentiginous melanomas and in a nodular melanoma. Overall, *NRAS* mutations were present in 29% of acral lentiginous melanomas. The two observed *NF1* mutations were a p.G453D missense and p.K1345* nonsense unreported mutations, detected in a nodular melanoma and in an acral lentiginous melanoma, respectively. 

We extended the mutational analysis of thirty cases to the main genes involved in melanoma pathogenesis using a custom-designed NGS panel (the genes included in the panel are reported in Supplementary Table S1). Overall, variants in at least one of 33 tested genes were identified in 26 of the 30 analyzed samples (87%) (mutations are reported in Supplementary Table S2). The *TP53* gene was mutated in 9 cases (30%; all acral lentiginous melanomas except for a nodular melanoma), frequently in association with *BRAF* mutations (5 out of 9 samples). *KIT* mutations were detected exclusively in three acral lentiginous melanomas (10%); in one case it was found in association with a *NF1* mutation and in another with a *NRAS* mutation. *TERT* promoter mutations were found in 4 cases (13%): two acral lentiginous melanomas also harbored a *BRAF* mutation, another a *NRAS* mutation, while a nodular melanoma harbored a *NF1* mutation. Among the genes that mutated in more than one AM and mutually exclusive with the TCGA genes, we found 3 *ARID1A* mutations in TWT samples: p.Trp1670* and p.Glu1291Asp mutations reported as pathogenic variants in the Catalogue of Somatic Mutations in Cancer (COSMIC https://cancer.sanger.ac.uk/cosmic, accessed on 10 March 2021) and p.Thr1514Met unreported variant. In addition, we found 3 *GRIN2A*, 2 *PTEN* and 2 *SF3B1* mutated cases. A single mutated AM was detected for *KMT2C*, *MITF*, *BAP1*, *NOTCH2*, *NOTCH1*, *DCC*, *DDX3X*, *ADAMTS18*, *PIK3CA*, *RB1*, *KRAS*, *PREX2*, *ERBB4*, *KDR*, and *MAP2K2* genes (Supplementary Table S2).

### 2.3. Molecular Classification and Clinicopathological Features

Associations between the TCGA molecular classification and the clinicopathological characteristics of patients are shown in Table 2. Statistical analyses compared *BRAF* vs. *NRAS* vs. TWT groups (the *NF1* group was excluded due to the small sample size). The TWT status was strongly associated with AM originating from the nail (*p* = 0.01), accounting for 4 out of 5 cases (80%). *BRAF*-mutated AM was related to a lower Breslow thickness (*p* = 0.02), a lower mitotic count (*p* < 0.01), and pT1-T2 stages (*p* = 0.03).

### 2.4. Genomic Copy Number Alterations

**Figure 1:**
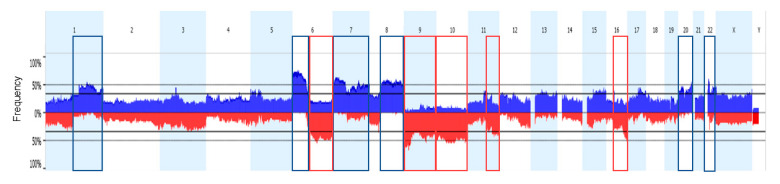
Whole-genome copy number profile. Aggregate frequency plot of copy number variations (CNVs) for 42 acral melanomas (AMs). Blue and red indicates gains and losses, respectively. The y-axis indicates the percentage of the samples in the cohort having an aberration at a specific point along the genome. Regions more frequently involved in gain and losses events are highlighted in blue and red boxes respectively.

**Figure 2:**
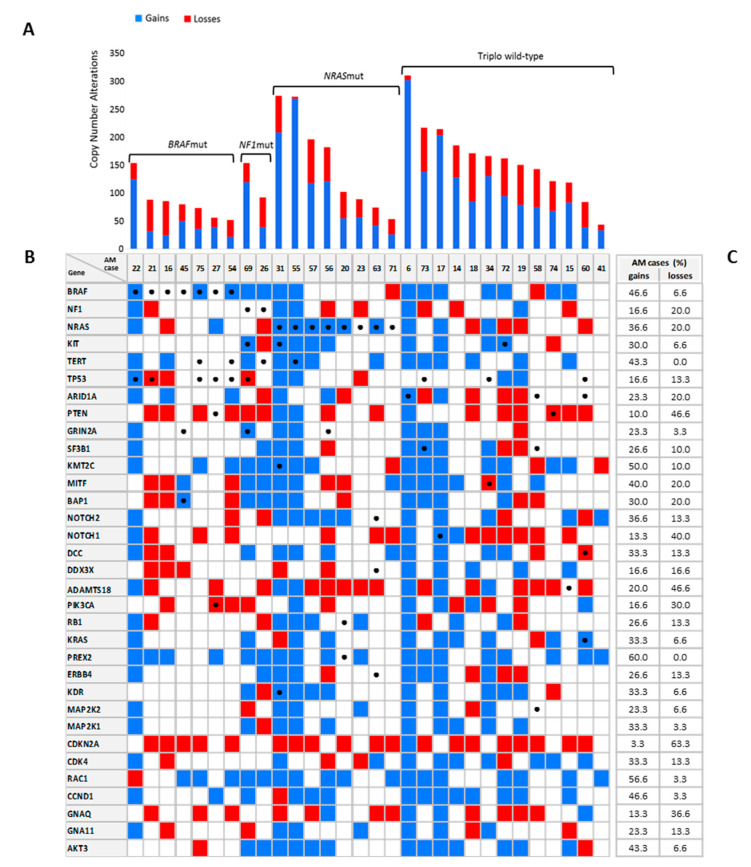
Copy number changes and mutations in melanoma driver genes in 30 AMs. (**A**) Copy number gains (blue) and losses (red) for each AM sample according to TCGA classification (*BRAF*/*NRAS*/*NF1* mutated and triple wild-type (TWT)). (**B**) Mutation and CNV analysis in 33 selected melanoma genes. For each AM sample, gene gains in blu and gene losses in red are reported. Genetic mutations are indicated by black dots. (**C**) Frequency of AM cases with gains and losses in the genes analyzed by next generation sequencing (NGS).

Somatic copy number variations (CNV) data were obtained using genome-wide single nucleotide polymorphism (SNP) arrays in 42 out of 48 analyzed AM samples. For all probes covering each locus, the average copy number was defined as a gain or loss if >2.0 or <2.0, respectively. In addition, a broader estimate concerning the percentage of genome changes was assessed, also considering the focal regions of loss of heterozygosis (LOH) and copy number (CN) neutral LOH. Overall, AMs showed a high percentage of genome change (median 34%, 2–91%) with a median of 121 CNVs (range 7–311). A high intertumoral heterogeneity with tetraploid samples and samples with only focal gains or losses were observed. Worth noting is that the AM (AM_43) with the lowest CNVs (*n* = 7) was the thin nevoid melanoma without ulceration and mitoses morphologically resembling a common nevus. On the other hand, the three cases presenting more than 80% of genome changes with more than 270 CNVs referred to locally advanced acral lentiginous melanomas with almost 65% of tetraploid tumor cells (data not shown) and with ulceration and number of mitoses/mm^2^ ≥ 4. In total, 66% of all identified CNVs were gains or amplifications mainly affecting both arms of the 7, 20, and 22 chromosomes and arm of the 1q, 6p, and 8q chromosomes (Figure 1). Among the sequenced genes, gains in *PREX2* (60% of cases), *RAC1* (57%), *KMT2C* (50%), *BRAF* (47%), *CCND1* (47%), *TERT* (47%), and *AKT3* (43%) genes were frequently observed. On the other hand, 34% of the CNVs were losses in the genomic region frequently spanning the 6q, 9, 10, 11q, and 16q chromosomes and more frequently involved in the *CDKN2A* (63% of cases), *PTEN* (47%) and *ADAMTS18* (47%) genes (Figure 2B,C).

### 2.5. Genomic Copy Number, Molecular, and Clinicopathological Features

Combining all CNVs into TCGA molecular subgroups (Figure 2A), *BRAF*-mutated cases (median 78, range 61–89) showed a significant lower count of aberrations compared to *NRAS* (median 103, range 90–197) and TWT cases (median 163; range 122–186; *p* = 0.03; Table 3). Moreover, this different distribution corresponded to a significant difference in the number of gains (*p* = 0.04) rather than losses (*p* = 0.28). Total CNVs was higher in thicker AM (*p* = 0.04), as well as in AM with ulceration (*p* = 0.03). In these latter cases, the gains were statistically more than in AM without ulceration (*p* = 0.02). Poor histologic prognostic factors, such as high Breslow thickness, high mitotic count, and presence of ulceration were associated with the percentage of changed genome (*p* = 0.003, 0.004, and 0.0002, respectively). Although not statistically significant due to the small sample size, the number of CNVs and the percentage of changed genome in pT3-4 cases (median CNVs 144; changed genome 42%) were almost twice those of pT1-2 cases (median CNVs 73; changed genome 22%).

### 2.6. Survival

The median follow-up of the 42 patients with molecular data was 41 months (IQR 17–68) and, during the study period, 20 patients had tumor recurrence (48%). The five-year DFS was 43% (95% CI 28–65%), while the five-year OS was 50% (95% CI 36–71%). The association between survival and clinically relevant factors is reported in Supplementary Table S3. High Breslow thickness and ulceration were associated with impaired DFS (HR 1.20, 95% CI 1.05 to 1.36, *p* = 0.008 and HR 6.83, 95% CI 1.13 to 29.90, *p* = 0.01, respectively). Older age (HR 1.05, 95% CI 1.02 to 1.09; *p* = 0.005) and high Breslow values (HR 1.26, 95% CI 1.13 to 1.40; *p* < 0.0001) were associated with impaired OS.

## 3. Discussion and Conclusions

AM is a rare subtype of malignant melanoma occurring in the non-hair-bearing skin of the palms, soles, and nail apparatus [1]. Despite a similar incidence among different ethnicities, AM is the commonest melanoma in Asian, African, and Hispanic people, given the low incidence of other cutaneous melanomas [1,2,3,14]. Most studies on AM were mainly conducted on these populations and European-derived people were often marginally included [15,16,17,18,19,20,21,22,23]. The present study attempted to add information for this last group by analyzing an Italian cohort of patients. Moreover, it focused on primary tumors, whereas most of the available molecular data on AM were derived from metastatic tissues which, therefore, may have accumulated more molecular alterations. However, this choice, along with that of using FFPE archival material, led to technical difficulties. Indeed, on the one hand, neoplastic material was frequently scarce because lesions were small. On the other, nucleic acids were often damaged by treatments with acid solutions used for bone decalcification and/or with sodium hydroxide solutions for nail softening present in the surgical samples and degraded by formalin fixation and long-lasting storage times. This affected the yield of extraction and amplifiability of the DNA in about 20% of initial samples. However, the remaining casuistry showed clinical features similar to those reported in the literature, such as presentation in old age, no gender predilection, main occurrence on the foot and 15% of subungual cases, and predominance of the acral lentiginous melanoma histotype, supporting its validity [1,10].

Overall, the current results confirmed the low mutational burden and the high percentage of genomic changes of AM, as previously reported [15,16,17,18,19,20,21,22,23]. Worth noting is that all the molecular alterations were detected in primary tumors, highlighting that they are probably early events in the development and progress of AM. Moreover, a gradient was observed in the accumulation of genomic structural alterations with a higher number of CNVs and percentage of changed genome in locally advanced AM.

According to the literature, the rate of *BRAF*-mutated AMs (35%) was lower than those reported for the other non-acral cutaneous melanomas [1,15,17,18]. However, it was higher than those usually found in this melanoma subtype, which rarely exceeds 20% of cases [15,16,24,25,27]. An explanation could be the fact that the present series only included Italian patients. Indeed, Yeh and colleagues highlighted that *BRAF* mutations were more common in European-derived patients, as also supported by the finding of 30% of AMs with *BRAF* mutations in a German cohort [16,23]. Of course, this result is of great importance from the therapeutic point of view since many cases are eligible for targeted therapy. And the fact that in more than 90% of cases the mutation is a p.V600E substitution is noteworthy from the diagnostic point of view, since it can also be detected by immunohistochemistry. *BRAF*-mutated AMs were related to more favorable histologic prognostic factors (thinner lesions with lower mitotic count) and harbored fewer copy number aberrations, similarly to melanomas occurring in non-chronically sun-damaged skin, in line with other studies [15,16]. In particular, *BRAF*-mutated cases were correlated with a lower frequency of gains in the *CCND1* locus compared to TWT AMs (29% and 61%, respectively), as reported by Curtin and colleagues [18]. No *BRAF* mutations were detected in subungual melanomas, confirming its rarity in this site [26,27]. The importance of this pathway in AM was also highlighted by the high percentage of cases with gains in the *BRAF* locus (47%).

The reported frequency of *NRAS* mutations in AM ranges from about 10% to 30% of cases, with markedly lower rates in melanomas of the nail apparatus [16,22,23,24,25,26,27]. In the present study, *NRAS* mutations were detected in 28% of AMs, none in subungual cases, and almost all in the acral lentiginous melanoma histotype. Although the most frequent *NRAS* mutation was in codon 61, as referred for the other non-acral cutaneous melanomas, about one-third of cases were mutated in codon 12. This is higher than usually reported in skin melanomas in a non-acral site, which is around 10%, but similar findings were already reported in AM [15,16,23,24,30]. Worth noting is that codon 12 of *NRAS* is not routinely investigated in skin melanoma because it rarely mutates. However, the present results suggest that this codon should be included in the mutational analysis of AM.

Only 5% of AMs were found to be *NF1*-mutated in this series, lower than the previously reported percentages [15,16,22,23] and *NF1* losses were observed in other 6 AMs. AMs with NF1 impairment have been related with worse histologic prognostic factors, an association that can neither be confirmed nor excluded by the present results due to the paucity of cases in this group [15]. It is noteworthy that NF1-defective *BRAF*, *NRAS*, and *KIT* wild-type melanoma cell lines have been shown to respond to MEK inhibitors in preclinical studies [31]. Moreover, *KIT* mutations were rare (10% of cases), a result significantly lower than reported in older studies where it seemed to be the prevalent molecular alteration in AM but in line with the more recent ones [16,17,22,23,28].

Among TWT samples, *ARID1A* mutations were found in three cases suggesting an important role in AM oncogenesis. ARID1A is a core member of the switch/sucrose non-fermentable (SWI/SNF) chromatin-remodeling complex, a machinery that provides the access of proteins to DNA, and its mutations have been frequently reported in many different types of cancers [32,33]. The pathogenicity of *ARID1A* mutations in melanoma was already reported in previous studies and aberrations in this pathway seem to occur in about 20% of AMs [19,34,35]. Shain and colleagues theorized that in the oncogenetic cascade of malignant melanoma an initiating mutation in *BRAF* or *NRAS* activates the mitogen-activated protein kinase (MAPK) signaling pathway and subsequent additional mutations hit other genes involved in tumor progression, such as the tumor suppressor gene *ARID1A* [36]. However, the MAPK pathway was not impaired by gene mutations among these three AMs and gains involving *BRAF* and *NRAS* loci were found in only a case, thus mutated-*ARID1A* might act also as driver. Indeed, ARID1A negatively regulates *TERT* expression and activity and its mutations might provide a survival advantage through telomere maintenance [33]. Moreover, *ARID1A* mutations and reduced copy number are reported to be negatively associated with patient survival and/or checkpoint therapy responses in multiple types of cancer [37]. In agreement with pan-cancer data sets from The Cancer Genome Atlas in the Skin Cutaneous Melanoma category (from https://www.cbioportal.org/, accessed on 10 March 2021, TCGA), we found that 23% and 20% of samples were characterized by *ARID1A* copy number gains and losses respectively. Future studies in a large cohort of treated patients may clarify if the *ARID1A* gene status can affect patient outcome in AM.

*TERT* promoter C>T transitions are found in about 70% of melanomas occurring on sun-exposed skin, suggesting a possible causative role of UV radiation [1,15,16,17,19,22,23,38,39,40,41]. AMs, instead, rarely harbor these mutations, thus excluding the UV-correlated pathogenesis [1,15,16,17,19,22,23,38,39,40,41]. Indeed, the peculiar body site and the thick stratum corneum overlying the tumor could act as protection from solar damage. Accordingly, *TERT* promoter mutations were detected only in 4 cases (13%). However, gains of the *TERT* locus were found in 47% of cases, highlighting the importance of this gene in the development and progression of melanoma. 

*TP53* mutation is considered a pivotal event in tumor progression, since it impairs the function of the gene protein product preventing the activation of apoptosis or the arrest of the cell cycle in response to DNA damage. However, its occurrence was less frequent in melanoma than in other cancers and it was rarely reported in AM [16,19,29]. Interestingly, in the present series, nine cases (accounting for 30% of analyzed samples) harbored *TP53* mutations and most were acral lentiginous melanomas. In the Catalogue of Somatic Mutations in Cancer, the *TP53* mutation rate is about 9% in AM, reaching 25% in the acral lentiginous melanoma subtype and similar results were found in other studies [42,43,44,45]. Besides the fact that only primary tumors were analyzed in this study, these data suggest that *TP53* mutations may be an early event in AM, allowing the accumulation of the high number of structural rearrangements and copy number changes usually found in this type of melanoma. Indeed, a high percentage of genome change was detected in the present AMs, especially in those cases with poor histologic prognostic features (thick lesion, presence of ulceration, and high mitotic count).

*PREX2* and *CDKN2A* were among the genes mainly affected by structural alterations in this series of AMs. Gains in the *PREX2* locus were detected in 60% of samples. PREX2 acts as a negative regulator of the PTEN tumor suppressor protein, thus promoting tumorigenesis by the activation of the PI3K signaling pathway [46,47]. Multiple mechanisms are implicated in *PREX2* dysregulation in melanoma. Indeed, *PREX2* gene amplification and rearrangements have been found [21]. Moreover, *PREX2* mutations were detected in about 15% of melanomas and immortalized human melanocytes expressing mutant *PREX2* have been reported to form tumors in mice [21]. Beside mutations, *PREX2* is altered via overexpression in various cancer types, including melanoma [48]. Indeed, overexpression of wild-type *PREX2* can promote tumor growth through the impairing of the PI3K pathway [47]. Altogether, these evidences suggest PREX2 as a key player in melanoma. *CDKN2A* is a well-known melanoma tumor-suppressor gene frequently deleted in all melanoma subtypes, particularly in mucosal and AM [18]. Indeed, *CDKN2A* losses were found in 63% of cases.

In summary, the present findings showed that several pathways (MAPK, PI3K, TP53, cell cycle, and SWI/SNF) may be impaired by genetic aberrations dominated by genomic structural variants. Survival analysis confirmed the prognostic value of classic histologic variables and ruled out any prognostic implication of molecular alterations which have, instead, predictive value of response to targeted therapy.

This study’s main strengths lie in the collection and analysis of primitive tumors only of homogeneous European-derived patients and in the use of solid and reliable molecular techniques. The main weaknesses of this investigation, instead, concern the retrospective setting, the limited number of cases analyzed, and the long-time span in which AMs were diagnosed. However, these features are shared with most studies on this topic and are mainly due to the rarity of this subtype of melanoma.

In conclusion, this study further supports most recent data regarding the frequencies and the variety of mutated genes and the high load of copy number variations in AM, highlighting their presence already at an early stage, mainly in tumors with poor histologic prognostic features. Moreover, it showed that the rate of *BRAF* mutations may be higher than previously reported in AMs of European-derived people and correlated with favorable prognostic factors such as low thickness, low mitotic count and an early-stages of patients with AM. 

## 4. Materials and Methods

### 4.1. Samples

Archival formalin-fixed and paraffin-embedded (FFPE) primary tumor samples of 74 patients diagnosed with AM during the period 2000–2019 at the Padua University Hospital, at the Veneto Institute of Oncology, or at the University Hospital of Turin were collected for this retrospective study. All cases were reviewed, the diagnoses confirmed in all instances by a pathologist according to the fourth edition of the World Health Organization classification of skin tumors, and the staging was updated to the 8th edition of the Union for International Cancer Control (UICC) TNM Classification of Malignant Tumors [9,49]. This study was approved by the institutional ethical review board of the Veneto Institute of Oncology IOV-I.R.C.C.S.

### 4.2. DNA Extraction

Tumor cell enrichment was performed to ensure a tumor cell content of >80%. Five consecutive 10 μm-thick sections were cut from each FFPE sample; tumor cells were then manually microdissected and collected in a sterile tube for DNA extraction. DNA was automatically isolated using the MagNA Pure Compact Nucleic Acid Isolation Kit I (Roche, Mannheim, Germany) on the MagNA Pure Compact instrument (Roche). DNA concentration was fluorometrically measured using a Qubit dsDNA HS assay (ThermoFisher Scientific, Waltham, MA, USA) on a Qubit Fluorometer 3.0 (ThermoFisher Scientific, Waltham, MA, USA). 

### 4.3. Sequencing Analysis

The DNA quality of ten AM cases only allowed *BRAF* codon 600 and *NRAS* codon 61 analysis by Sanger sequencing or by high sensitivity commercial real-time PCR-based assays, namely EasyPGX ready BRAF and EasyPGX ready NRAS (Diatech Pharmacogenetics, Ancona, Italy), in accordance with the manufacturer’s protocol. Sanger sequencing was performed by amplifying exon 15 of *BRAF* and exon 3 of *NRAS*. The amplified products were purified with Illustra GFX 96 PCR Purification Kit (GE Healthcare, Buckinghamshire, UK) and sequenced using the BigDye Terminator v1.1 Cycle Sequencing Kit (ThermoFisher Scientific). After purification with a BigDye XTerminator Purification Kit (ThermoFisher Scientific), sequences were analyzed on the 96-capillary automatic sequencer AB3730xl Genetic Analyzer (ThermoFisher Scientific). 

The DNA of thirty AM cases was suitable for NGS sequencing. In accordance with the manufacturer’s protocol, 100 ng of dsDNA was used to construct the library using a TruSeq Custom Amplicon Low Input Kit (Illumina, San Diego, CA, USA) in combination with a 33 gene custom-designed panel (Illumina, San Diego, CA, USA; Supplementary Table S1). The included genes were selected based on literature-based evidences [35,45,50,51]. Briefly, samples were subjected to dual-strand amplicon-based PCR library preparation and the quality of the libraries was then estimated using a High Sensitivity DNA Analysis Kit (Agilent Technologies, Santa Clara, CA, USA) on a Bionalyzer 2100 system (Agilent Technologies, Santa Clara, CA, USA). Subsequent sequencing of pooled libraries was performed in the MiSeq sequencing platform (Illumina, San Diego, CA, USA) using V3 reagents. 

Data analysis, including alignment to the hg19 human reference genome, variant calling and annotation was done using a customized somatic pipeline on SOPHiA DDM-v4 software (Sophia Genetics, Saint-Sulpice, Switzerland). The selection of variants was based on a minimum coverage of 400X, minimum frequency of mutated allele of 15% and previous classification as pathogenic, likely pathogenic or variant of unknown significance on common databases of somatic variants (COSMIC database and cBioPortal). In order to exclude common SNPs, we only considered variants (splicing variants, promoter variants and exonic missense and nonsense variants) with a reported frequency of ≤0.05. Intronic and synonymous variants were not included in the analysis. The detected variants were then validated by Sanger Sequencing, as described above. 

### 4.4. Copy Number Variation (CNV) Analysis

From forty-two AM cases, 80 ng of dsDNA was analysed for genome-wide copy number variations (CNV) using the OncoScan CNV Assay (ThermoFisher Scientific, Waltham, MA, USA) on an Affymetrix SNP-array platform (ThermoFisher Scientific, Waltham, MA, USA), in accordance with the manufacturer’s protocol. This assay is based on molecular inversion probe technology specifically designed to handle limited amounts of highly degraded FFPE-extracted DNA. 

Raw probe signal intensities (CEL files) obtained in this manner were processed using OncoScan Console software (ThermoFisher Scientific, Waltham, MA, USA). Normal FFPE controls from the OncoScan assay Kit were used to calculate log2 ratio and B-allele frequencies (BAF). Copy-number aberrations, percentage of genome changed, and sample ploidy were identified by OncoScan Nexus Copy Number 3 (Biodiscovery, Hawthorne, CA, USA) using the TuScan segmentation algorithm. Log2 ratios for each marker were calculated relative to the reference signal profile. For two copies of alleles, the log graph should be centered around 0 on the Y axis: genomic regions were classified as having gains or losses if the Log2 ratios exceeded or fell below this threshold.

The genomic region of LOH or CN neutral LOH were included in the “percentage of genome changed” overviewing the percentage of sample genome aberrations.

### 4.5. Statistical Analysis

Continuous data (age at diagnosis, Breslow thickness and number of mitoses) were summarized as mean and standard deviation (SD), or median and interquartile range (IQR). The association between variables was assessed using the Mann-Whitney test, Kruskal-Wallis test, Spearman correlation coefficient, Chi Square test, and Fisher’s exact test, as appropriate. Survival estimates were calculated using the Kaplan-Meier method. The association between clinically relevant variables and progression-free and overall survival (PFS and OS, respectively) was evaluated using Cox regression models, with effect sizes expressed as the hazard ratio (HR) with a 95 per cent confidence interval (95% CI). All tests were two-sided and a p-value less than 0.05 was considered statistically significant. Statistical analysis was performed using R 4.0 (R Foundation for Statistical Computing, Vienna, Austria) [52].

## Figures and Tables

**Table 1:** Clinical and pathological characteristics of the 48 molecularly analyzed patients.

Clinical and Pathological Variables	
*Age at diagnosis (yrs)*	
Mean (SD)	71.2 (13.2)
Range	47–88
*Breslow thickness (mm)*	
Mean (SD)	5.1 (3.7)
Range	0.7–19.2
*Mitoses (number/mm^2^)*	
Mean (SD)	8.3 (8.4)
Range	0–45
*Gender (n, %)*	
Male	24 (50%)
Female	24 (50%)
*Tumor site (n, %)*	
Hand	3 (6%)
Foot	38 (79%)
Nail	7 (15%)
*Histotype (n, %)*	
ALM	40 (83%)
NM	6 (13%)
SSM	1 (2%)
Nevoid melanoma	1 (2%)
*pT classification (n, %)*	
T1	1 (2%)
T2	6 (13%)
T3	16 (33%)
T4	23 (47%)
NE	2 (5%)
*Ulceration (n, %)*	
Absent	18 (37%)
Present	28 (58%)
NE	2 (5%)
*Regression (n, %)*	
Absent	42 (87%)
Present	4 (8%)
NE	2 (5%)
*Lymphovascular Invasion (n, %)*	
Absent	30 (62%)
Present	15 (32%)
NE	3 (6%)
*Perineural invasion (n, %)*	
Absent	34 (71%)
Present	11 (23%)
NE	3 (6%)

SD = standard deviation; ALM = acral lentiginous melanoma; NM = nodular melanoma; SSM = superficial spreading melanoma; NE = not evaluable.

**Table 2:** Clinical and pathological characteristics of the 40 patients with mutational data according to the TCGA classification.

Clinical and Pathological Variables	BRAF	NF1	NRAS	TWT	*p*-Value ^a^
*N patients*	14	2	11	13	-
*Age, yrs: median (IQR)*	70 (57–85)	50; 86	74 (65–76)	70 (61–79)	0.99
*Breslow thickness, mm: median (IQR)*	2.4 (1.7–4.0)	6.9; 8.6	3.7 (3.3–5.3)	4.6 (3.7–7.0)	0.02
*Mitoses, number/mm^2^: median (IQR)*	2 (1–5)	10; 15	4 (4–5)	7 (5–20)	0.007
*Tumor site: n (%)*					0.01
Hand/foot	14 (40%)	1 (3%)	11 (31%)	9 (26%)	
Nail	0 (0)	1 (20%)	0 (0)	4 (80%)	
*Histotype: n (%)*					0.60 ^b^
ALM	11 (32%)	1 (3%)	10 (29%)	12 (36%)	
Nevoid melanoma	1 (100%)	0 (0)	0 (0)	0 (0)	
NM	1 (25%)	1 (25%)	1 (25%)	1 (25%)	
SSM	1 (100%)	0 (0)	0 (0)	0 (0)	
*pT classification: n (%) * ^c^					0.03
T1-T2	5 (83%)	0 (0)	0 (0)	1 (17%)	
T3-T4	8 (25%)	2 (7%)	11 (34%)	11 (34%)	
*Ulceration: n (%) * ^c^					0.26
Absent	7 (44%)	0 (0)	6 (37%)	3 (19%)	
Present	6 (27%)	2 (9%)	5 (23%)	9 (41%)	
*Regression: n (%) * ^c^					0.55
Absent	11 (31%)	2 (5%)	11 (32%)	11 (32%)	
Present	2 (67%)	0 (0)	0 (0)	1 (33%)	
*Lymphovascular Invasion: n (%) * ^c^					0.90
Absent	8 (31%)	2 (8%)	7 (27%)	9 (34%)	
Present	4 (36%)	0 (0)	4 (36%)	3 (28%)	
*Perineural invasion: n (%) * ^c^					0.37
Absent	11 (39%)	1 (6%)	8 (26%)	8 (29%)	
Present	1 (11%)	1 (11%)	3 (33%)	4 (45%)	

^a^*p*-value calculated comparing *BRAF* mutated vs. *NRAS* mutated vs. triple wild-type (TWT) cases; the 2 *NF1* mutated cases were excluded; ^b^ *p*-value calculated comparing ALM vs. Nevoid/NM/SSM cases; ^c^ cases without evaluable data were excluded (Table 1, NE cases); IQR: interquartile range; ALM = acral lentiginous melanoma; NM = nodular melanoma; SSM = superficial spreading melanoma.

**Table 3:** Clinicopathological characteristics and molecular data of the 42 patients with CNV analysis.

Clinical and Pathological Variables	Total CN Alterations		Gains		Losses		Percentage of Changed Genome	
	Summary Measures	*p*-Value	Summary Measures	*p*-Value	Summary Measures	*p*-Value	Summary Measures	*p*-Value
*Age (yrs) (n = 42)*, *Spearman correlation coefficient*	0.21	0.19	0.28	0.07	0.15	0.33	0.06	0.70
*Breslow thickness (mm) (n = 40)*, *Spearman correlation coefficient*	0.31	0.04	0.23	0.16	0.29	0.07	0.45	0.003
*Mitoses (number/mm^2^) (n = 41)*, *Spearman correlation coefficient*	0.24	0.13	0.17	0.30	0.29	0.07	0.44	0.004
*TCGA classification*,								
BRAF (*n* = 12), median (IQR)	78 (61–89)	0.03 ^a^	39 (27–56)	0.04 ^a^	30 (20–51)	0.28 ^a^	29 (19–36)	0.23 ^a^
NF1 (*n* = 2), median (IQR)	93; 155		40; 121		34; 53		55; 57	
NRAS (*n* = 11), median (IQR)	103 (90–197)		58 (43–122)		47 (32–62)		30 (23–67)	
TWT (*n* = 13), median (IQR)	163 (122–186)		86 (76–131)		53 (36–68)		35 (33–68)	
*Tumor site*		0.99		0.93		0.89		0.64
Hand/foot (*n* = 36), median (IQR)	113 (81–175)		69 (39–123)		47 (30–63)		33 (24–68)	
Nail (*n* = 6), median (IQR)	133 (100–158)		73 (47–91)		53 (22–64)		46 (33–66)	
*Histotype*		0.09		0.07		0.34		0.67
ALM (*n* = 35), median (IQR)	151 (88–178)		84 (42–124)		53 (30–67)		34 (25–68)	
Nevoid/NM (*n* = 7), median (IQR)	85 (68–99)		40 (33–49)		46 (26–57)		42 (21–54)	
*Ulceration*		0.03		0.02		0.36		0.0002
Absent (*n* = 15), median (IQR)	89 (65–145)		40 (30–78)		53 (22–61)		25 (20–32)	
Present (*n* = 25), median (IQR)	155 (99–199)		86 (51–138)		53 (30–67)		53 (33–70)	
*Lymphovascular Invasion*		0.31		0.77		0.59		0.38
Absent (*n* = 26), median (IQR)	113 (74–171)		64 (40–122)		42 (28–66)		34 (24–57)	
Present (*n* = 13), median (IQR)	155 (98–197)		86 (36–119)		61 (36–62)		37 (30–69)	
*Perineural invasion*		0.46		0.58		0.42		0.47
Absent (*n* = 28), median (IQR)	114 (75–165)		64 (38–124)		42 (25–66)		39 (25–69)	
Present (*n* = 11, median (IQR)	168 (96–185)		86 (49–121)		61 (45–62)		31 (26–54)	

^a^*p*-value calculated comparing *BRAF* mutated vs. *NRAS* mutated vs. TWT cases; ALM = acral lentiginous melanoma; NM = nodular melanoma.

## Data Availability

Data will be available upon request.

## Supplementary Materials

The following are available online at https://www.mdpi.com/article/10.3390/ijms22083826/s1.
