# Supplementary material for: The Molecular Landscape of Primary Acral Melanoma: A Multicenter Study of the Italian Melanoma Intergroup (IMI)
Source: Int J Mol Sci. 2021 Apr 7;22(8):3826. doi: 10.3390/ijms22083826 (PMC8067752; doi:10.3390/ijms22083826)
Supplement: Supplementary file 1 [file ijms-22-03826-s001.zip › Elefanti_Table S1_revised.pdf]

Table S1: Custom panel used for genetic testing of acral melanomas. It consists in amplicons of median size range approximately of 150 bp, within the main genes involved in melanoma pathogenesis .

Chromosome	Gene	Transcript	sequenced exons^	Sequence
chr1	AKT3	ENST00000263826.9	2	NC_000001.11
chr1	NOTCH2	ENST00000256646.6	1-3, 30-31, 34	NC_000001.11
chr1	NRAS	ENST00000369535.4	2-3	NC_000001.11
chr1	ARID1A	ENST00000324856.11	2, 7, 16-20	NC_000001.11
chr2	ERBB4	ENST00000342788.8	3-28	NC_000002.12
chr2	SF3B1	ENSG00000115524	1-25	NC_000002.12
chr3	MITF	ENST00000352241.8	2-10	NC_000003.12
chr3	PIK3CA	ENST00000263967.3	2-10, 14-21	NC_000003.12
chr3	BAP1	ENST00000460680.5	3-17	NC_000003.12
chr4	KIT +rs17084733	ENST00000288135.5	2-6, 9, 11, 13-14, 17-19, 21	NC_000004.12
chr4	KDR	ENST00000263923.4	7-16, 20-26	NC_000004.12
chr5	TERT	ENST00000310581.9	promoter -300 exon 1	NC_000005.10
chr7	MLL3 (KMT2C)	ENST00000262189.10	6-10, 14, 18, 45-49, 58	NC_000007.14
chr7	BRAF	ENST00000288602.10	11 e 15	NC_000007.14
chr7	RAC1	ENST00000356142.4	2	NC_000007.14
chr8	PREX2	ENST00000288368.4	6, 8, 19, 32, 34, 38	NC_000008.11
chr9	NOTCH1	ENST00000277541.6	3, 6, 11, 17-23, 26-31, 34	NC_000009.12
chr9	GNAQ	ENST00000286548.8	4 e 5	NC_000009.12
chr9	CDKN2A	ENST00000498124.1	1-3	NC_000009.12
chr10	PTEN	ENST00000371953.7	1-9	NC_000010.11
chr11	CCND1	ENST00000227507.2	1-5	NC_000011.10
chr12	CDK4	ENST00000257904.10	2	NC_000012.12
chr12	KRAS	ENST00000311936.7	2 e 3	NC_000012.12
chr13	RB1	ENST00000267163.4	2-23	NC_000013.11
chr15	MAP2K1	ENST00000307102.9	2-6	NC_000015.10
chr16	GRIN2A	ENST00000396573.6	1-14	NC_000016.10
chr16	ADAMTS18	ENST00000282849.9	3-23	NC_000016.10
chr17	NF1	ENST00000358273.8	3-12, 17-34, 38, 39, 43-54	NC_000017.11
chr17	TP53	ENST00000269305.8	1-11	NC_000017.11
chr18	DCC	ENST00000442544.6	1-29	NC_000018.10
chr19	GNA11	ENST00000078429.8	4 e 5	NC_000019.10
chr19	MAP2K2	ENSG00000126934	1-11	NC_000019.10
chrX	DDX3X	ENST00000399959.6	2-4, 7-14	NC_000023.11

^splice site sequences at the intron-exon junction were included
