# Supplementary material for: The Molecular Landscape of Primary Acral Melanoma: A Multicenter Study of the Italian Melanoma Intergroup (IMI)
Source: Int J Mol Sci. 2021 Apr 7;22(8):3826. doi: 10.3390/ijms22083826 (PMC8067752; doi:10.3390/ijms22083826)
Supplement: Supplementary file 1 [file ijms-22-03826-s001.zip › Elefanti_Table S2_revised.pdf]

Table S2: NGS sequencing results

ID AM_case	Histotype	refGenome	Gene	RefSeq	genome_position	nucleotide variant	amino acid variant	coding_consequence	Cosmic ID	FATHMM prediction
AM_06	ALM	GRCh37/hg19	ARID1A	NM_006015	27100077	c.3873G>T	p.Glu1291Asp	missense	COSM7408869	Pathogenic
AM_15	ALM	GRCh37/hg19	ADAMTS18	NM_199355	77359816	c.1979G>A	p.Ser660Asn	missense	COSM8112067	Pathogenic
AM_16	ALM	GRCh37/hg19	BRAF	NM_004333	140453136	c.1799T>A	p.Val600Glu	missense	COSM476	Pathogenic
AM_17	ALM	GRCh37/hg19	NOTCH1	NM_017617	139391833	c.6358C>T	p.Arg2120Cys	missense	/	VUS
AM_20	ALM	GRCh37/hg19	NRAS	NM_002524	115258747	c.35G>A	p.Gly12Asp	missense	COSM564	Pathogenic
AM_20		GRCh37/hg19	PREX2	NM_024870	69058536	c.4180C>T	p.Arg1394Trp	missense	COSM3750133	Pathogenic
AM_20		GRCh37/hg19	RB1	NM_000321	49030387	c.1862G>A	p.Arg621His	missense	COSM4962401	Pathogenic
AM_21	ALM	GRCh37/hg19	BRAF	NM_004333	140453136	c.1799T>A	p.Val600Glu	missense	COSM476	Pathogenic
AM_21		GRCh37/hg19	TP53	NM_000546	7577593	c.686_687delGT	p.Cys229Tyrfs*10	frameshift	COSM43648	na
AM_22	ALM	GRCh37/hg19	BRAF	NM_004333	140453136	c.1799T>A	p.Val600Glu	missense	COSM476	Pathogenic
AM_22		GRCh37/hg19	TP53	NM_000546	7574029	c.998G>A	p.Arg333His	missense	COSM6008899	Pathogenic
AM_23	ALM	GRCh37/hg19	NRAS	NM_002524	115256528	c.183A>T	p.Gln61His	missense	COSM585	Pathogenic
AM_26	NM	GRCh37/hg19	TERT	NM_001193376	1295250	c.-146C>T	/	intergenic	/	
AM_26		GRCh37/hg19	NF1	NM_000546	29533355	c.1358G>A	p.Gly453Asp	missense	na	
AM_27	ALM	GRCh37/hg19	PIK3CA	NM_006218	178936029	c.1571G>A	p.Arg524Lys	missense	COSM53245	Pathogenic
AM_27		GRCh37/hg19	BRAF	NM_004333	140453136	c.1799T>A	p.Val600Glu	missense	COSM476	Pathogenic
AM_27		GRCh37/hg19	TP53	NM_000546	7578388	c.542G>A	p.Arg181His	missense	COSM10738	Pathogenic
AM_27		GRCh37/hg19	PTEN	NM_000314	89712014	c.632G>T	p.Cys211Phe	missense		
AM_31	ALM	GRCh37/hg19	NRAS	NM_002524	115258748	c.34G>T	p.Gly12Cys	missense	COSM562	Pathogenic
AM_31		GRCh37/hg19	KDR	NM_002253	55964922	c.2315C>T	p.Ala772Val	missense	COSM172826	Pathogenic
AM_31		GRCh37/hg19	KMT2C	NM_170606	151962134	c.1173C>A	p.Cys391*	nonsense	COSM230133	Pathogenic
AM_31		GRCh37/hg19	KIT	NM_000222	55593613	c.1679T>A	p.Val560Asp	missense	COSM1257	Pathogenic
AM_34	ALM	GRCh37/hg19	MITF	NM_198159	70008501	c.1091G>A	p.Arg364His	missense	COSM2784799	Pathogenic
AM_34		GRCh37/hg19	MITF	NM_198159	70008489	c.1079G>A	p.Arg360Gln	missense	COSM4120058	Pathogenic
AM_34		GRCh37/hg19	TP53	NM_000546	7574003	c.1024C>T	p.Arg342*	nonsense	COSM11073	Pathogenic
AM_45	ALM	GRCh37/hg19	BAP1	NM_004656	52437753	c.1408G>A	p.Gly470Arg	missense	COSM9312294	na
AM_45		GRCh37/hg19	BRAF	NM_004333	140453135	c.1799_1800delTGinsAC	p.Val600Asp	missense	COSM308550	Pathogenic
AM_45		GRCh37/hg19	GRIN2A	NM_000833	9857574	c.3827C>G	p.Ala1276Gly	missense	COSM107888	Pathogenic
AM_54	ALM	GRCh37/hg19	BRAF	NM_004333	140453136	c.1799T>A	p.Val600Glu	missense	COSM476	Pathogenic
AM_54		GRCh37/hg19	TERT	NM_001193376	1295250	c.-146C>T	/	intergenic	/	
AM_54		GRCh37/hg19	TP53	NM_000546	7578212	c.637C>T	p.Arg213*	nonsense	COSM10654	Pathogenic
AM_55	ALM	GRCh37/hg19	NRAS	NM_002524	115256530	c.181C>A	p.Gln61Lys	missense	COSM580	Pathogenic
AM_55		GRCh37/hg19	TERT	NM_001193376	1295228	c.-124C>T		intergenic	/	
AM_56	ALM	GRCh37/hg19	NRAS	NM_002524	115258744	c.38G>A	p.Gly13Asp	missense	COSM573	Pathogenic
AM_56		GRCh37/hg19	GRIN2A	NM_000833	9858173	c.3228C>A	p.Asn1076Lys	missense	COSM9177401	Pathogenic
AM_57	ALM	GRCh37/hg19	NRAS	NM_002524	115256528	c.183A>C	p.Gln61His	missense	COSM586	Pathogenic
AM_58	ALM	GRCh37/hg19	ARID1A	NM_006015	27101259	c.4541C>T	p.Thr1514Met	missense	na	
AM_58		GRCh37/hg19	SF3B1	NM_012433	198283262	c.466A>G	p.Met156Val	missense	na	
AM_58		GRCh37/hg19	MAP2K2	NM_030662	4101088	c.634G>T	p.Asp212Tyr	missense	na	
AM_60	NM	GRCh37/hg19	ARID1A	NM_006015	27102084	c.5010G>A	p.Trp1670*	nonsense	COSM5490625	Pathogenic
AM_60		GRCh37/hg19	KRAS	NM_004985	25380315	c.143G>A	p.Gly48Glu	missense	na	
AM_60		GRCh37/hg19	TP53	NM_000546	7579344	c.343C>T	p.His115Tyr	missense	COSM4429612	Pathogenic
AM_60		GRCh37/hg19	DCC	NM_005215	51056945	c.4266G>T	p.Gln1422His	missense	na	
AM_63	ALM	GRCh37/hg19	NOTCH2	NM_024408	120548162	c.205G>A	p.Glu69Lys	missense	na	
AM_63		GRCh37/hg19	NRAS	NM_002524	115256529	c.182A>G	p.Gln61Arg	missense	COSM584	Pathogenic
AM_63		GRCh37/hg19	ERBB4	NM_001042599	212576777	c.1122T>G	p.His374Gln	missense	COSM9359678	Pathogenic
AM_63		GRCh37/hg19	DDX3X	NM_001356	41206111	c.1616-1G>A	p.?	splice_acceptor	na	
AM_69	ALM	GRCh37/hg19	KIT	NM_000222	55599342	c.2468A>G	p.Tyr823Cys	missense	COSM18682	Pathogenic
AM_69		GRCh37/hg19	GRIN2A	NM_000833	9857094	c.4307A>G	p.Asn1436Ser	missense	COSM7370288	Pathogenic
AM_69		GRCh37/hg19	NF1	NM_000267	29576060	c.4033A>T	p.Lys1345*	nonsense	na	
AM_69		GRCh37/hg19	TP53	NM_000546	7577542	c.739A>G	p.Asn247Asp	missense	COSM45005	Pathogenic
AM_71	NM	GRCh37/hg19	NRAS	NM_002524	115256529	c.182A>G	p.Gln61Arg	missense	COSM584	Pathogenic
AM_72	ALM	GRCh37/hg19	KIT	NM_000222	55594221	c.1924A>G	p.Lys642Glu	missense	COSM1304	Pathogenic
AM_73	ALM	GRCh37/hg19	SF3B1	NM_012433	198267483	c.1874G>T	p.Arg625Leu	missense	COSM110695	Pathogenic
AM_73		GRCh37/hg19	TP53	NM_000546	7578406	c.524G>A	p.Arg175His	missense	COSM10648	Pathogenic
AM_74	ALM	GRCh37/hg19	PTEN	NM_000314	89692851	c.335T>C	p.(Leu112Pro)	missense	COSM5106	Pathogenic
AM_75	ALM	GRCh37/hg19	TERT	NM_001193376	1295250	c.-146C>T	/	intergenic	/	
AM_75		GRCh37/hg19	BRAF	NM_004333	140453136	c.1799T>A	p.Val600Glu	missense	COSM476	Pathogenic
AM_75		GRCh37/hg19	TP53	NM_000546	7578212	c.637C>T	p.Arg213*	nonsense	COSM10654	Pathogenic
