# Supplementary material for: The Molecular Landscape of Primary Acral Melanoma: A Multicenter Study of the Italian Melanoma Intergroup (IMI)
Source: Int J Mol Sci. 2021 Apr 7;22(8):3826. doi: 10.3390/ijms22083826 (PMC8067752; doi:10.3390/ijms22083826)
Supplement: Supplementary file 1 [file ijms-22-03826-s001.zip › Elefanti_Table S3.pdf]

Table S3. Survival analysis of the 42 patients with molecular data

**Clinical and pathological variables**	**DFS**		**OS**	
	**HR (95% CI)**	**p-value**	**HR (95% CI)**	**p-value**
Age, years (n=42)	1.01 (0.98 to 1.04)	0.54	1.05 (1.02 to 1.09)	**0.005**
Breslow (n=40)	1.20 (1.05 to 1.36)	**0.008**	1.26 (1.13 to 1.40)	**<0.0001**
Mitosis, mm^2 (n=41)	1.01 (0.96 to 1.06)	0.66	1.04 (0.99 to 1.08)	0.07
Total CN aberrations (n=42)	1.00 (0.99 to 1.01)	0.82	1.00 (0.99 to 1.01)	0.66
Gains (n=42)	1.00 (0.99 to 1.01)	0.97	1.00 (0.99 to 1.01)	0.82
Losses (n=42)	1.00 (0.98 to 1.03)	0.56	1.00 (0.98 to 1.02)	0.41
Percentage of changed genome (n=42)	1.00 (0.98 to 1.02)	0.37	1.00 (0.98 to 1.02)	0.67
TGCA classification:				
- BRAF (n=14)	1.35 (0.43 to 4.27)	0.51	0.39(0.13 to 1.18)	0.10
- NRAS (n=11)	0.66 (0.19 to 2.30)	0.61	0.42 (0.13 to 1.37)	0.15
- TWT (n=13)	Reference		Reference	
Tumor site:				
- Hand/foot (n=36)	Reference	0.63	Reference	0.30
- Nail (n=6)	0.61 (0.08 to 4.59)		1.94 (0.56 to 6.70)	
Histotype:				
- ALM (n=35)	Reference	0.52	Reference	0.28
- Nevoid Melanoma, NM (n=7)	0.67 (0.19 to 2.28)		0.50 (0.15 to 1.72)	
pT:				
- T1-T2 (n=5)	0.35 (0.05 to 2.64)	0.31	0.55 (0.12 to 2.42)	0.43
- T3-T4 (n=35)	Reference		Reference	
Ulceration:				
- Absent (n=15)	Reference	**0.01**	Reference	0.12
- Present (n=25)	6.83 (1.56 to 29.90)		2.22 (0.81 to 6.07)	
Lymphovascular Invasion:				
- Absent (n=26)	Reference	0.12	Reference	0.33
- Present (n=13)	2.15 (0.82 to 5.61)		1.55 (0.63 to 3.75)	
Perineural invasion:				
- Absent (n=28)	Reference	0.68	Reference	0.28
- Present (n=11)	0.80 (0.28 to 2.28)		1.67 (0.66 to 4.19)	

[a]Hazard ratio (95% confidence interval)
Regression status was not included in this analysis due to the small number of patients with regression (n=3).
